# Insights on Genomic and Molecular Alterations in Multiple Myeloma and Their Incorporation towards Risk-Adapted Treatment Strategy: Concise Clinical Review

**DOI:** 10.1155/2017/6934183

**Published:** 2017-11-08

**Authors:** Taiga Nishihori, Kenneth Shain

**Affiliations:** ^1^Department of Blood and Marrow Transplantation, H. Lee Moffitt Cancer Center and Research Institute, Tampa, FL, USA; ^2^Department of Malignant Hematology, H. Lee Moffitt Cancer Center and Research Institute, Tampa, FL, USA

## Abstract

Although recent advances in novel treatment approaches and therapeutics have shifted the treatment landscape of multiple myeloma, it remains an incurable plasma cell malignancy. Growing knowledge of the genome and expressed genomic information characterizing the biologic behavior of multiple myeloma continues to accumulate. However, translation and incorporation of vast molecular understanding of complex tumor biology to deliver personalized and precision treatment to cure multiple myeloma have not been successful to date. Our review focuses on current evidence and understanding of myeloma biology with characterization in the context of genomic and molecular alterations. We also discuss future clinical application of the genomic and molecular knowledge, and more translational research is needed to benefit our myeloma patients.

## 1. Introduction

Multiple myeloma remains an all but incurable terminally differentiated B cell malignancy. We have long understood that multiple myeloma evolves from a premalignant condition of terminally differentiated B cells. Many, if not all, myeloma patients are considered to have harbored a premalignant monoclonal gammopathy of undetermined significance (MGUS) that progresses to smoldering or asymptomatic multiple myeloma and ultimately to symptomatic multiple myeloma requiring therapeutic intervention [1]. This “symptomatic” threshold is characterized as the worsening of myeloma associated multiorgan dysfunction and may be associated with altered genetic profiles [2–4]. Typical symptomatic presentation of multiple myeloma includes hypercalcemia, renal failure, anemia, and bone lesions (i.e., lytic lesions) and/or fractures, collectively known as “CRAB.” The recent explosion of available antimyeloma agents targeting particular pathways or antigens including various proteasome inhibitors (such as bortezomib, carfilzomib, and ixazomib), immunomodulatory agents, pan-histone deacetylase inhibitor, and anti-CS1 and anti-CD38 monoclonal antibodies, as well as widespread adaptation of high-dose chemotherapy followed by autologous hematopoietic cell transplantation (HCT) has led to growing optimism for the treatment of myeloma and indeed has shown overall improvement in therapeutic outlook and survivals [1, 5]. Despite these remarkable achievements, long term control of myeloma can only be achieved in minority of patients in part due to inevitable development of clonal evolution and therapy resistance stemming from a number of factors including influences on the genome including the stress of therapy and interactions of myeloma cells and bone marrow microenvironment in the context of environment mediated drug resistance (EMDR) [6, 7]. Clonal evolution results in genotypically dynamic and, therefore, phenotypically dynamic myeloma cell populations with varying sensitivities to therapy. As such, there is a critical need to be able to characterize the molecular aspects of disease both at diagnosis (prognostic) and throughout the course disease to best allocate care and understand the best use of available antimyeloma agents to control this heterogeneous disease.

The last decade has observed rapid growth of knowledge pertaining to genomic and molecular characterization of multiple myeloma which was made possible due to technological advancements. Myeloma characterization has evolved from traditional microscopic examination and conventional flow cytometry to genomic analysis using methods for gene expression profiling (GEP), proteomics, and beyond. Most clinical risk stratification relies on standard karyotypic analysis and fluorescent in situ hybridization (FISH) which comprise the bulk of high-risk definition but emerging data exist to incorporate GEP data into prognostication of myeloma. In this review, we focus on the review of current knowledge with regard to risk stratification and incorporation of GEP and further genomic and molecular information to future myeloma treatment strategies.

## 2. Known Genetic Alterations Associated with High-Risk Myeloma

For a decade we have developed different molecular tools to characterize a classic set of myeloma-related genetic alterations involving duplication of odd numbered chromosomes, translocations of immunoglobulin heavy chain (IgH) enhancers and oncogenes, and deletions of tumor suppressors that aid us in prognostic risk stratification. The anomalies aid in the parsing of patients into low- and high-risk stages/classifications. Complete and comprehensive characterization of cytogenetic changes in multiple myeloma has been reviewed elsewhere and it is beyond the scope of this brief review [8]. The dichotomy in risk dictates that standard (low-risk) risk is defined by the presence of hyperdiploidy (multiple copies of odd numbered chromosomes) and translocations involving IgH enhancers with D-type cyclins (t(6;14) and t(11;14)). Here, we focus on a number of representative chromosomal alterations commonly seen in high-risk multiple myeloma, a disease state that remains to be the bane of myeloma therapy. The t(4;14) (p16;q32) has been shown to be associated with poor survival [9, 10]. This translocation results in increased expression of fibroblast growth factor receptor 3 (FGFR3) and multiple myeloma SET (MMSET) domain [11, 12]. Translocations involving* maf *genes have been found in minority of myeloma and these derive from IgH rearrangements with a locus in chromosome 16, most commonly t(14;16)(q32;q23). The t(14;16) or other 16q abnormalities may occur in conjunction with chromosome 13 deletion, another common genetic alteration in multiple myeloma portending increased risk [9]. One study showed deletion of 13q in 85% of myeloma patients with t(4;14) and 92% of t(14;16) suggesting strong correlations between deletion 13q and 14q32 rearrangements [13]. These chromosomal changes involving 4 and 16 are thought to be primary high-risk genetic events [14]. Further, studies have also suggested that loss of chromosome 16 and/or increased expression of the* FOPNL* gene at 16p13 may be linked to poorer outcomes in myeloma [15].

Additionally, monoallelic deletion of 17p13, the locus of tumor suppressor gene* p53*, confers aggressive clinical course, extramedullary disease, and inferior survival [10, 14]. Majority of chromosome 1 abnormalities involve rearrangements located in the pericentromeric regions and chromosome 1 abnormalities have been relatively recently added to major negative prognostic factor in myeloma including transplant-eligible patients [16–18]. Both 1q gain (or 1q21 amplification) and 1p loss could occur as chromosome 1 abnormalities. Gene expression signature for high-risk disease is also enriched disproportionately for genes located in chromosome 1 supporting the importance of chromosome 1 abnormalities in risk stratification [16]. Biologic and genetic underpinning of chromosome 1 abnormalities has been debated but some initial studies suggested possible association of* CKS1B* [19]. Deletion of chromosome 13 or 13q when encountered in conventional karyotyping (but not by FISH) is also considered high-risk.

These recurrent high-risk cytogenetic changes may coexist with other cytogenetic alterations and the combination of these features may modulate the risk status. For example, trisomies of chromosomes 3 and 5 in addition to known high-risk cytogenetics may lessen the overall high-risk significance and trisomy of chromosome 21 may worsen the outcomes [20, 21]. These represent specific examples; however, with our growing ability to molecularly characterize the genome and the expressed genome we anticipate that we may be able to provide even better risk stratification even when faced with coexistent complex molecular changes in a personalized context and possibly dictate therapy [22].

## 3. Current Risk Stratification and Clinical Staging of Multiple Myeloma

Prognostic staging of myeloma has long been limited to assessment of laboratory values (albumin and beta-2 microglobulin) as defined by the International Staging System (ISS) [23]. With a parallel risk stratification based on myelomatous molecular changes and in recognition of the importance of these factors collectively in portending risk for myeloma patients, the International Myeloma Working Group (IMWG) published their consensus statement on myeloma risk classification and reported that combination of (a) ISS stage II or III and (b) t(4;14) or deletion of 17p13 would be considered high-risk myeloma on the basis of limited overall survival (OS) of approximately 2 years [24]. Further, a similar incorporation of this molecular high-risk classification was used in establishing the Revised-ISS (R-ISS) with the addition of t(14;16) as well as elevated lactate dehydrogenase (LDH) and those with R-ISS stage III myeloma are projected to have median OS of 43 months highlighting the significant adverse impact of certain genomic alterations in myeloma over a simple unidimensional measurement of tumor burden as the disease stage [25]. IMWG also posed therapeutic questions for the high-risk group by asking the research community to examine novel therapeutic strategies such as allogeneic hematopoietic cell transplantation or immunotherapy to improve the outcomes [24].

## 4. Gene Expression Profiling in Myeloma

High-throughput genomic tools such as GEP have been extensively investigated in recent years to predict patient outcomes. A pioneering effort in multiple myeloma with GEP was made by the group at University of Arkansas for Medical Sciences (UAMS) where they identified an initial 70-gene signature allowing a new definition of high-risk myeloma by GEP under the treatment platform of Total Therapy II which was followed by a reduced number of 17-gene model providing prognostic information [16]. Of note, this high-risk gene profile seems to be enriched for genes located in chromosome 1. A recent logistic regression analysis of the 70-gene score using the data from UAMS Total Therapies II and III by van Laar et al. showed that the 70-gene prognostic risk score is continuously associated with increased risk of 5-year relapse and death reconfirming the importance of this gene score [26]. The Intergroupe Francophone du Myélome (IFM) also identified 15 genes that would predict poor prognosis in multiple myeloma [27]. GEP studies suggested that there are possibly up to 8 different molecular subtypes in multiple myeloma and opened our eyes to the complexity and difficulty of molecular characterization of myeloma [28, 29]. Indeed, there are several other GEP-based prognostic signatures that were developed including centrosome index [30], genes with homozygous deletion [31], human myeloma cell lines with IL-6 stimulation [32], proliferation index [33], and others [34, 35]. Interestingly, these individual GEP signatures demonstrate minimal overlap amongst them; however, this is likely expected as each GEP signature might be associated with different but important myeloma tumor biology (such as proliferation) related genes thus a minimum number of such genes would be represented in each signature. Although each GEP signature has been validated using different independent data sets, there is no universal agreement on the GEP signature(s) to be considered as a gold standard of high-risk definition as each of these GEP signature was developed in the different treatment and baseline risk context.

The gene expression microarray has become the most commonly used technology for genomic investigation in oncology which is also robust and has good interlaboratory agreement [36]. There are increasing number of clinical applications using GEP in medicine for more refined classifications of a tumor that was not previously possible or for treatment decisions; however, the latter lags behind likely due to multitude of unresolved issues. For example, there are several GEP signatures reported for multiple myeloma as above and these different signatures do not necessarily correlate with one another. Some investigators have reported their attempts to incorporate GEP information into clinical practice with the aim to provide a precision patient care in multiple myeloma. Meißner et al. built the GEP-report (GEP-R) using an open-source software developed in R using Affymetrix microarrays [37]. GEP-R integrated GEP-based (both UAMS- and IFM-scores) and conventional prognostic factors such as proliferation, ISS stage, t(4;14), and expression of prognostic target genes* (AURKA*,* IGF1R)* and developed one risk stratification termed HM-metascore [37]. The HM-metascore distinguished 3 different risk groups, low-risk, medium-risk versus high-risk with 6-year survival rates of 89.3%, 60.6%, and 18.6%, respectively. This system allows automated interpretation of Affymetrix U133 Plus 2.0 gene expression profiles which has been in use in some European centers for both routine clinical practice and clinical trials. The larger issue might be related to the fact that high-risk GEP signature has yet to be linked with specific treatment options. For example, Hose et al. identified varying frequencies of aurora-A and aurora-B expression in myeloma samples using Affymetrix DNA microarrays and presence of aurora-A expression was associated with inferior event-free and OS [38]; however, aurora kinase inhibitors have not been commercially available to translate this finding into clinical practice. Additionally, it is still possible that novel GEP signature may be identified or defined in the future which may be of clinical interest to predict specific novel therapeutic interventions especially for immunotherapeutic approaches and we expect that the landscape of high-risk myeloma GEP signatures would evolve over time. To date, GEP has not facilitated the rationale selection of antimyeloma agents and RNA sequencing may replace microarray-based GEP in the future. More comprehensive analysis of differential RNA splicing and isoform expression are expected to inform myeloma characterization.

## 5. Moving towards Risk-Adapted Therapeutic Approaches in Myeloma Using Genomic Information

Though risk stratification is commonly performed, there are currently no widely accepted consensus risk-adapted approaches for multiple myeloma treatment based on genomic information. National Comprehensive Cancer Network (NCCN) guidelines on multiple myeloma provide list of potential combination chemotherapy options but they do not suggest modifications of treatment strategy based on risk status [39]. Group of investigators at Mayo Clinic published myeloma treatment guidelines which suggest the utilization of myeloma risk stratification to generate risk-adapted therapy approaches termed mSMART (https://www.msmart.org/) [40]. mSMART version 2.0 classifies deletion of 17p, t(14;16) and t(14;20) as high-risk and t(4;14) and 1q gain as intermediate-risk myeloma. They suggest to consider carfilzomib/lenalidomide/dexamethasone induction in transplant-eligible high-risk myeloma population. Bortezomib-based maintenance is considered after autologous HCT in both intermediate- and high-risk population with consideration of carfilzomib-based maintenance in high-risk myeloma.

With more antimyeloma agents in hand in the past few years, data exist to consider differential usage of target specific therapeutic approaches which would be expected to evolve over time. Some studies suggested that bortezomib-based regimen may abrogate or reduce the negative survival impact of t(4;14) and t(14;16) or possibly deletion of 17p [41–44]. In addition, at least 2 reports suggested that incorporation of bortezomib before and after autologous HCT might overcome some of the poor prognostic significance posed by deletion of 17p13 [45, 46]. Of note, though thalidomide has fallen out of favor for maintenance therapy after the availability of lenalidomide, the Medical Research Council (MRC) IX study showed that patients with high-risk disease (including gain of 1q, deletion of 1p32, t(4;14), t(14;16), and t(14;20), and deletion of 17p) had worse OS with thalidomide maintenance [47]. Emerging evidence indicates that newer antimyeloma agents such as carfilzomib and pomalidomide may preferentially improve outcomes in high-risk myeloma patients [48–50].

While only limited data from randomized controlled studies are available to inform the development of universal consensus on risk-adapted treatment strategies, the risk stratification schema could serve as useful frameworks for the development of rational design and treatment selection of specific myeloma therapeutic clinical trials. For instance, Bone Marrow Transplant Clinical Trial Network (BMT CTN) has designed a multicenter randomized placebo-controlled trial evaluating a novel allogeneic HCT clinical trial using fludarabine, melphalan, and bortezomib conditioning and posttransplant ixazomib maintenance specifically targeting high-risk myeloma patients (https://clinicaltrials.gov/ct2/show/NCT02440464). Investigators at Johns Hopkins have also launched a phase 2 clinical trial evaluating the role of marrow infiltrating lymphocytes (MILs) in high-risk myeloma in the context of high-dose melphalan and autologous HCT (https://clinicaltrials.gov/ct2/show/NCT01858558) [51]. Though not universally accepted as a high-risk definition, some clinical trials use a commercially available GEP signature as one of the study eligibility criteria for high-risk myeloma. It is expected that many future novel immunotherapeutic approaches including chimeric antigen receptor (CAR) T cell therapy would be designed using the risk stratification to overcome poor overall outcomes in high-risk myeloma patients.

## 6. Growing Knowledge of Genomic and Molecular Information in Myeloma Characterization

Various DNA-based high-throughput technologies (also termed next generation sequencing (NGS)) including whole-genome sequencing (WGS) and whole-exome sequencing (WES) have been developed and have become the common genomic tools to evaluate the origin and evolution of cancer cells over the past 2 decades. These technologies have also been applied to expand and better understand the biology of multiple myeloma. Chapman et al. performed the initial study of WGS in 38 myeloma patients [52] which was followed by at least 3 other large scale NGS studies in myeloma to evaluate the mutational landscape [53, 54]. The study by Chapman et al. showed 10 statistically significant protein-coding mutations at a false discovery rate of ≤ 0.10 including* NRAS*,* KRAS*,* FAM46C*,* DIS3*,* TP53*,* CCND1*,* PNRC1*,* ALOX12B*,* HLA-A*, and* MAGED1* but frequencies were low [52]. Of note,* BRAF* mutations (G469A, K601N, and V600E) in myeloma were discovered albeit at low frequency (7 out of 161 patients = 4%) [52]. Walker et al. performed WES in over 400 myeloma patients through Myeloma XI trial and reported somewhat overlapping significant mutations in 15 genes [55]. Taken together, genes involved in mitogen-activated protein kinase (MAPK) pathway such as* NRAS*,* KRAS*, and* BRAF* (RAS pathway 43%) and those in the nuclear factor (NF)-kappa B pathway (17%) are recurrently mutated in myeloma and these are likely important drivers of myelomagenesis [55].

These studies showed wide variety of mutations with varying frequencies and the significance of individual gene mutations in relation to survival outcomes might be difficult to discern due to the complex subclonal mixtures in one patient. However, in one study, a complex subclonal structure and clusters of subclonal variants correlated with a high-risk of relapse and death [53]. The study also identified new candidate genes including truncations of* SP140*,* LTB*, and* ROBO1* and clustered missense mutations in* EGR1* [53]. In another study, presence of* TP53*,* ATM*,* ATR*, and* CCND1* mutations was associated with inferior OS in multivariate analysis [55]. It is conceivable that emerging mutational and molecular information may be incorporated into the future risk stratification and prognostication of myeloma patients.

Moving further into personalized treatment decision making, this mutational profiling may guide therapeutic choice in the future with ever increasing number of available antimyeloma agents or nonmyeloma specific but pathway specific drugs. In a study of 133 patients with relapsed myeloma treated with single-agent bortezomib, a targeted sequencing of a panel of 41 known oncogenes and tumor suppressor genes was performed [56]. Mutually exclusive mutations of* BRAF* and* RAS* were seen in closer to a half of patients and mutations of* NRAS* (but not* KRAS*) were associated with lower response rate to bortezomib [56]. On the other hand, mutations in* IRF4 *which are believed to be the downstream of immunomodulatory drug target of cereblon seem to be associated with better survival [55]. These studies uncovered actionable mutations in multiple myeloma, and coupled with explosion of various pathways and targeted inhibitors there is a growing optimism to hope for mutation specific adapted treatment strategies becoming reality in the near future.

However, detailed characterization of mutational landscape in multiple myeloma also disclosed complexity in predicting and interpreting the NGS data due to higher number of coexisting clones and subclonal mutations. Clonal evolution in myeloma has been documented to involve linear evolution, differential clonal response, and branching evolution [53]. Careful delineation of subclonal population with sequential targeted sequencing would likely be required to accurately predict treatment outcomes using mutational information. Another challenge may involve coexistence of downstream activating mutations in the same pathway which may render some of the targeted therapies ineffective. Additionally, we have to acknowledge that genes are the essential component of a phenotype though the latter may also be influenced by other external or environmental factors. Epigenetic modifications such as histone methylation as well as the stresses of therapy and myeloma ecosystem (bone marrow microenvironment) could also potentially influence myeloma clinical behavior (i.e., clonal evolution) and further research is needed to investigate these possibilities. Therefore, there is an urgent need to develop comprehensive informatics to combine all genomic, epigenetic, and other molecular information to accurately prognosticate myeloma patients' survival and to rationally select target specific therapies based on genomic and epigenetic alterations.
